# Long-distance gene flow and adaptation of forest trees to rapid climate change

**DOI:** 10.1111/j.1461-0248.2012.01746.x

**Published:** 2012-04

**Authors:** Antoine Kremer, Ophélie Ronce, Juan J Robledo-Arnuncio, Frédéric Guillaume, Gil Bohrer, Ran Nathan, Jon R Bridle, Richard Gomulkiewicz, Etienne K Klein, Kermit Ritland, Anna Kuparinen, Sophie Gerber, Silvio Schueler

**Affiliations:** 1 INRA, UMR1202 Biodiversité Gènes et CommunautésCestas, F-33610, France; 2 Université de Bordeaux, UMR1202 Biodiversité Gènes et CommunautésTalence, F-33410, France; 3 Université Montpellier 2 CNRS, UMR5554, Institut des Sciences de l’EvolutionF-34095 Montpellier Cedex 05, France; 4 Department of Forest Ecology and Genetics, Forest Research Centre (CIFOR)INIA, 28040 Madrid, Spain; 5 ETH, Department of Environmental SciencesUniversitätstrasse 16 8092 Zürich, Switzerland; 6 Department of Civil, Environmental & Geodetic Engineering, Ohio State UniversityColumbus, OH 43210, USA; 7 Movement Ecology Laboratory, Department of Ecology, Evolution and Behavior, The Alexander Silberman Institute of Life Sciences, The Hebrew University of Jerusalem, Edmond J. Safra CampusJerusalem 91904, Israel; 8 School of Biological Sciences, University of BristolBristol, BS8 IUG, UK; 9 School of Biological Sciences and Department of Mathematics, Washington State UniversityPullman, Washington 99164, USA; 10 INRA, UR Biostatistiques & Processus Spatiaux 546F-84914 Avignon, France; 11 Department of Forest Sciences, University of British ColumbiaVancouver, BC V6T 1Z4, Canada; 12 Ecological Genetics Research Unit, Department of Biosciences, University of HelsinkiHelsinki FI-00014, Finland; 13 Federal Research and Training Centre for Forests, Natural Hazards and LandscapeSeckendorf-Gudent-Weg 8, 1131 Wien, Austria

**Keywords:** Adaptation, climate change, forest trees, gene flow, selection

## Abstract

Forest trees are the dominant species in many parts of the world and predicting how they might respond to climate change is a vital global concern. Trees are capable of long-distance gene flow, which can promote adaptive evolution in novel environments by increasing genetic variation for fitness. It is unclear, however, if this can compensate for maladaptive effects of gene flow and for the long-generation times of trees. We critically review data on the extent of long-distance gene flow and summarise theory that allows us to predict evolutionary responses of trees to climate change. Estimates of long-distance gene flow based both on direct observations and on genetic methods provide evidence that genes can move over spatial scales larger than habitat shifts predicted under climate change within one generation. Both theoretical and empirical data suggest that the positive effects of gene flow on adaptation may dominate in many instances. The balance of positive to negative consequences of gene flow may, however, differ for leading edge, core and rear sections of forest distributions. We propose future experimental and theoretical research that would better integrate dispersal biology with evolutionary quantitative genetics and improve predictions of tree responses to climate change.

## Introduction

**Figure 1:**
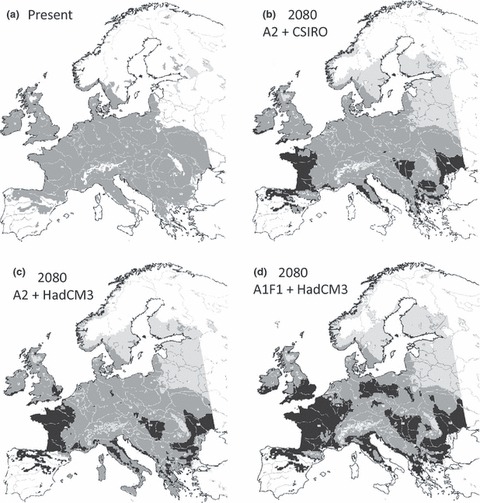
Predicted shifts of *bioclimatic envelopes* of sessile oak (*Quercus petraea)* in Europe (according to Thuiller 2003). Predicted *bioclimatic envelopes* of sessile oak in 2080, assuming that correlations between present distribution [panel (a), dark grey area] and climatic data are maintained. Black areas would no longer be climatically suitable for sessile oak in 2080, whereas light grey areas indicate areas that would become climatically suitable by 2080. Predictions were made according to different IPCC models of greenhouse gas emissions (GG) and climatic changes (CC) (Solomon *et al.* 2007). Panel (b): GG is A2 and CC is CSIRO2; panel (c): GG is A2 and CC is HadCM3; panel (d): GG is A1F1 and CC is HadCM3. Straight lines limiting the eastern margins of the bioclimatic envelopes are due to data limitations and do not represent predictions of envelopes.

Although evidence of climate change and its impact on world’s biota is steadily increasing, so are our concerns about the biological or human-mediated capacities of species and populations to cope with these changes (Solomon *et al.* 2007). Such concerns are more acute for sedentary and long-lived organisms such as trees, which may not be able to rapidly track favourable conditions through migration. Furthermore, trees constitute a large ecologically and economically important functional group of woody plants that dominate many terrestrial ecosystems in regions where the most pronounced climate changes are projected to occur. For example, the near-surface temperature is expected to shift northwards in mean rates of 110–430 m year^−1^ during the twenty-first century for Mediterranean, temperate and boreal forests, the major forest biomes in mid and high latitudes (Loarie *et al.* 2009). Niche modelling under various climatic and greenhouse emission predictions suggests that *bioclimatic envelopes* (Glossary) for forest trees will shift northwards in North America (Iverson *et al.* 2008) and north-eastwards in Europe (Thuiller 2003; Fig. 1). The estimated shift distance varies from 300 to 800 km within one century depending on the climate or greenhouse gas emission scenario (Mc Kenney *et al.* 2007), with considerable variation across both models and species.

Shifts in *bioclimatic envelopes* are likely to generate not only potential extinction and recolonisation, but also large reorganisation of genetic diversity within the species range if divergent or locally adapted populations respond in different ways. Indeed, the large *bioclimatic envelope* of many forest trees hides a collection of highly differentiated populations and genotypes with contrasting adaptation to local climate (Savolainen *et al.* 2007). In particular, climatic tolerances of local populations are typically much narrower than the whole species climatic envelope (Box 1). With climate change, environments change continuously and the optimal sets of adaptations maximising fitness under local conditions may shift accordingly. The evolutionary responses of populations can then be pictured as a race where populations are tracking the moving optima both in time and space (Pease *et al.* 1989; Polechova *et al.* 2009). Migration and adaptation are often perceived as alternative responses to these challenges (Aitken *et al.* 2008) because evolution allows populations to adapt to novel conditions without migrating, whereas migration lets populations track favourable conditions without evolving. However, range shifts and adaptation will likely occur simultaneously (e.g. Cwynar & MacDonald 1987). Indeed, seed dispersal allows colonisation of new favourable habitat, but, both seed and pollen dispersal in trees also affect the spread of genetic variation within the range.

**Box 1 Provenance tests and norms of reaction**

*Provenance tests* are common garden experiments that usually consist of a large number of populations planted by forest geneticists in multiple replicates over decades (Morgenstern 1996). They provide crucial information on the level of genetic variation within and between populations for fitness related traits, resulting from a balance of divergent selection across populations, gene flow, and random genetic drift. Extensive surveys of genetic diversity and variation have been conducted in these experiments and indicate that (1) extant populations harbour large levels of genetic variation continuously replenished by extensive gene flow (Hamrick *et al.* 1992); (2) *adaptive traits* exhibit high levels of population differentiation, despite gene flow, as a result of strong divergent selection (Savolainen *et al.* 2007); (3) clinal patterns of population differentiation along climatic or geographical variables are congruent across species suggesting systematic adaptive responses to directional selection, particularly for phenological traits (Mimura & Aitken 2007a); and (4) the extant distribution of between vs. within population differentiation for fitness related traits has developed rapidly following post-glacial recolonisation and is not the legacy of ancient population structure (Kremer *et al.* 2010). Furthermore, when replicated *provenance tests* were established, *reaction norms* of populations can be constructed that visualise their response across a wide range of environmental conditions (Rehfeldt *et al.* 1999, 2002). *Reaction norms* of fitness-related traits follow generally quadratic functions. Panel (a) illustrates the reactions norms of two populations of *Pinus contorta* (Rehfeldt *et al.* 1999) for height at age 20 (Pop 1, Udy Creek coming from 53° N and Pop 2, Muncho Lake coming from latitude 59.1° N). Δ accounts for the difference between the climate of the site where the population stems from (dotted line) and the optimal climate corresponding to the site where the population exhibits the highest value for height (bold line). Such *reaction norms* show that the climatic tolerances of individual *provenances* are narrower than the whole species climatic envelope, that climatic optima for growth differ among local populations, correlating with their climate of origin, and that climatic tolerance only partially overlap between *provenances*. These tests also suggest that populations located at the extremities of the natural distribution inhabit climates that are suboptimal for their growth and development. For population 2 (coming from latitude 59.1° N), as illustrated in (a), the optimal climate (2.5 °C) is warmer than the climate of its geographic source (−2.5 °C), whereas for extreme southern populations the opposite pattern occurs [panel (b)]. The overall picture is a negative correlation between Δ and the latitude of origin of the population as shown in panel (b) by the example in the case of *Pinus contorta* (Rehfeldt *et al.* 1999). Note on panel (b) that populations located near the horizontal dashed line are optimally suited to their local environment. This pattern is consistent with theoretical predictions that asymmetric gene flow from the core to peripheral populations increases maladaptation at the edges of the natural distribution (Garcia-Ramos & Kirkpatrick 1997; Kirkpatrick & Barton 1997). Figures modified from Rehfeldt *et al.* 1999 with permission of the Publisher.

**Figure:**
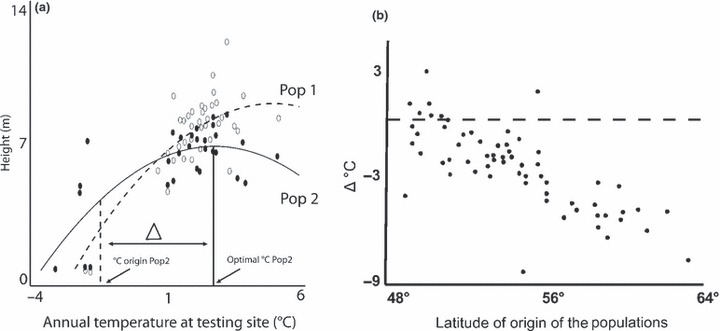

Trees are characterised by their particular life history, combining long-generation time (allowing divergent strategies at different life stages) and the capacity for long dispersal distances through pollen and seeds. Given the anticipated intensity and directionality of climatic change, do trees have the adaptive capacity to respond and how will gene flow affect that response? Valuable insights into these issues are provided by the retrospective analysis of adaptation in tree populations during the climate change that occurred following the last glaciations (Petit *et al.* 2008). Decades-old common garden experiments of forest trees in the Northern hemisphere suggest that an interaction between divergent selection across contrasting environments and large pollen flow allowed forest trees populations to adapt to past changing environments (Kremer *et al.* 2010). Whether interaction between gene flow and selection will be as efficient in the future remains unclear, as the predicted rates of environmental changes might exceed historical ones, and as gene flow may be constrained today by fragmentation.

We here critically examine the hypothesis that long-distance (LD) gene flow could compensate for the long-generation time of trees, facilitating evolutionary change in a shifting climate, by reviewing both data and theory, about the extent of gene flow in forest trees and its evolutionary consequences. We first review the recent literature on LD pollen and seed dispersal in trees and show that their spatial range is frequently comparable to the predicted scale of habitat shifts within one generation. We then review the theoretical predictions and experimental evidence for the effects that such LD gene flow could have on local adaptation in trees. We conclude that the positive effects of gene flow may often dominate its negative effects, although regional variation may influence the balance of those effects. We finally elaborate on the theoretical and experimental approaches that should be implemented to improve our ability to predict the scale and distribution of gene flow effects on forest ecosystems in the context of climate change.

## How far do seeds and pollen disperse in trees?

**Figure 2:**
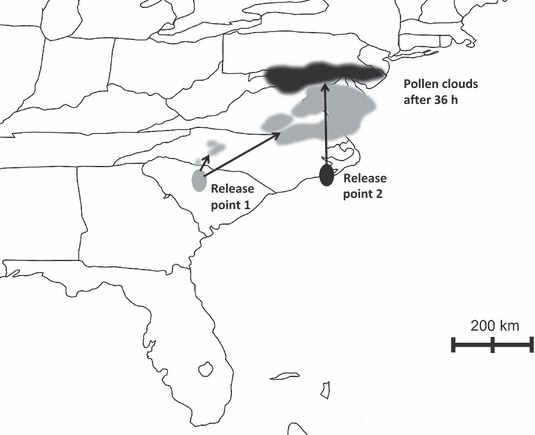
Virtual long-distance pollen dispersal of *Pinus taeda.* Virtual pollen release, using the Regional Atmospheric Modeling System (RAMS). The experimental settings are described in Bohrerova *et al.* (2009). Pollen was released from two locations, in North Carolina outer banks (black point) and South Carolina (grey point), at 6:00 pm, 27 March 2006 corresponding with observed peak of pollen release nearby. The dispersing pollen plumes (black – NC; grey – SC) are shown at 6:00 am, 36 h after the release. The wind was moderate, mainly toward the northeast. Mortality due to UV and vapour pressure deficit is resolved based on bench-scale observations. The pollen in the image, 36 h after release is *c.* 40% viable.

Gene flow in plants is mediated by both seed and pollen dispersal, which vary greatly among species (Ennos 1994). Seed and pollen dispersal, however, have distinct effects on the rate of demographic spread, and the rate at which genes move across the range of a species. The spatial scale of *effective propagule dispersal* (Glossary) in trees depends on a variety of physical and biological processes that determine the amount and availability of pollen and seeds, their movement, their viability before and during movement, and the probability of successful pollination leading to viable seed and the seedling establishment rates. Different combinations of these components may yield *effective dispersal* (Glossary) distances spanning from a few centimetres to thousands of kilometres (Nathan *et al.* 2008), generally following markedly leptokurtic patterns. Aerobiological studies show that airborne tree pollen (both viable and non-viable) has the potential to be transported in substantial amounts over hundreds to thousands of kilometres (Table 1, Fig. 2). Documented dispersal distances of viable pollen (yet prior to successful fertilisation) are however about one order of magnitude shorter, up to 600 km (Table 1). Documented distances of effective pollen dispersal (when pollination led to successful mating) are of lower magnitude, up to 100 km (Table 1). Documented wind-driven effective seed dispersal is up to a few kilometres (Table 1), thus about two orders of magnitude shorter than effective pollen dispersal. Although animal-mediated seed dispersal can reach a scale of tens of kilometres, pollen dispersal distances are in general considerably longer than that of seeds, and especially in wind-driven dispersal systems.

**Table 1:** Examples of observed LD pollen and seed dispersal in trees (more than 3 km for pollen and 1 km for seeds). The table is arranged first by propagule type (pollen or seed), then by vector type (wind, insects…) and dispersal type (potential, viable or effective), and finally alphabetically by species name

	Dispersal system					Dispersal distance		
Species	Propagule	Vector	Location	Method	Type*	Maximum	Proportion ≥ threshold†	Reference
*Betula* spp.	Pollen	Wind	Potential	Central-North-Eastern Europe	Aerobiologic and phenological analysis	1000 km		Siljamo *et al.* 2008
*Pinus banksiana* and *Picea glauca*	Pollen	Wind	Potential	Canada	Aerobiologic analysis	3000 km		Campbell *et al.* 1999
*Pinus sylvestris*	Pollen	Wind	Viable	Northern Europe	Aerobiologic and phenological analysis	600 km		Varis *et al.* 2009
*Pinus taeda*	Pollen	Wind	Viable	Eastern North America	Aerobiologic analysis	40 km		Williams 2010
*Cecropia obtusifolia*	Pollen	Wind	Effective	Central America	Genetic paternity analysis		10% > 14 km	Kaufman *et al.* 1998
*Fraxinus excelsior*	Pollen	Wind	Effective	Scotland	Genetic parentage analysis		25–35% > 3 km	Bacles *et al.* 2006
*Pinus sylvestris*	Pollen	Wind	Effective	Spain	Genetic mixture analysis		4.3% > ∼100 km	Robledo-Arnuncio 2011
*Quercus robur*	Pollen	Wind	Effective	Eastern Europe	Genetic parentage analysis		35% > 80 km	Buschbom *et al.* 2011
*Populus trichocarpa*	Pollen	Wind	Effective	Western North America	Genetic paternity analysis		5% > ∼5–10 km	Slavov *et al.* 2009
*Ficus* spp.	Pollen	Insects	Effective	Central America	Genetic parental reconstruction	14 km (isolated mother trees)		Nason *et al.* 1998
*Ficus sycomorus*	Pollen	Insects	Effective	Namibia	Genetic paternity analysis	165 km		Ahmed *et al.* 2009
*Sorbus domestica*	Pollen	Insects	Effective	Central Europe	Genetic paternity analysis		∼1% > 12–16 km	Kamm *et al.* 2009
*Swietenia humilis*	Pollen	Insects	Effective	Central America	Genetic paternity analysis		40–80% ≥ 4 km (in small fragments)	White *et al.* 2002
*Fraxinus excelsior*	Seed	Wind	Effective	Scotland	Genetic parentage analysis	1.4 km	46–53% > 3 km	Bacles *et al.* 2006
*Annona glabra*	Seed	Birds	Potential	Australia	Empirically based simulations of vector movements and seed passage time	5.2 km	1% > 4 km	Westcott *et al.* 2008
*Xylopia hypolampra* and 7 other species	Seed	Birds	Potential	Cameroon	Empirically based simulations of vector movements and seed passage time	6.9 km		Holbrook & Smith 2000
*Ficus carica* and *Morus alba*	Seed	Bats	Potential	Israel	Empirically based simulations of vector movements and seed passage time	20 km	17% > 1 km	Tsoar *et al.* 2011
*Tamarindus indica*	Seed	Elephants	Potential	Myanmar (Burma)	Empirically based simulations of vector movements and seed passage time	5.4 km	50% > 1.2 km	Campos-Arceiz *et al.* 2008
*Duroia duckei* and 2 other species	Seed	Fish	Potential	Peru	Empirically based simulations of vector movements and seed passage time	5.5 km	5% > 1.7 km	Anderson *et al.* 2011
*Prunus mahaleb*	Seed	Vertebrates	Potential	Spain	Genetic maternal analysis		33% > 1500 m	Jordano *et al.* 2007
*Sorbus domestica*	Seed	Vertebrates	Effective	Central Europe	Genetic paternity analysis	12.2 km		Kamm *et al.* 2009

* Three types are distinguished: ‘potential’ dispersal is the distance dispersed by a propagule at any, commonly unknown, condition; ‘viable’ is the same as ‘potential’ but excluding non-viable propagules; cases of ‘effective’ dispersal are the pollen that gave rise to seeds, or seeds that established, yielding seedlings, saplings or young/adult plants.

† The proportion (%) of propagules dispersed to equal or greater distances than the specified threshold. The threshold distances were defined by the authors of each study, often arbitrarily or according to features of the study landscape and/or populations.

Detecting mating events over hundreds of kilometres is experimentally very difficult and many biological processes take place (viability, phenology and pollen competition) between pollen release and fertilisation. Similarly, quantifying the multiple demographic and dispersal parameters affecting seed dispersal and subsequent establishment is still hard to accomplish (but see Nathan *et al.* 2011). These experimental constraints and biological reality both explain the disparities between reported potential and *effective dispersal* distances. Their relative contributions remain unresolved and addressing this point will require innovation (see section ‘Develop new methods to trace pollen and seeds’). Interactions between physical and biological processes influencing LD dispersal (LDD) and the way these will be affected in a changing climate are particularly poorly understood. The local environment around the release point of the propagule and the conditions at which the propagule was released can have strong effects of either decreasing or increasing the rate and extent of LD propagule transport (Bohrer *et al.* 2008; Wright *et al.* 2008). Also important is the interaction between weather patterns that drive extreme LDD, and the conditions that affect survival during the dispersal event, particularly in pollen, where a migration event may span many hours and days (Schueler *et al.* 2005).

In that respect, predicting how climate change itself may modify patterns of gene flow in the future remains a formidable challenge. Projected changes such as warmer atmosphere (Kuparinen *et al.* 2009), or higher fecundity, earlier maturation, and changes (reduction or increase) in surface wind speed (Nathan *et al.* 2011) can be implemented into mechanistic models of plant spread in future environments. Although this approach facilitates assessing the upper and lower bounds of future gene flow and population spread, it is unlikely to provide accurate predictions for a particular species and system, due to the large uncertainty about key hard-to-measure parameters, such as the spatial patterns of phenological schedules for pollen and of post-dispersal survival of seeds. Moreover, genetic variation in traits affecting dispersal of both pollen and seeds is common in plants and, accordingly, plant dispersal traits have been observed to rapidly evolve in response to environmental change, especially in the context of range expansion (Darling *et al.* 2008). In particular, increased frequency of traits facilitating seed dispersal have been found in recently founded populations, at the expanding edge of the range, whereas the converse trend was found in fragmented southern populations at the rear end (Riba *et al.* 2009). Identifying how global change (including climate change) will alter selection pressures acting on dispersal is therefore crucial to predict the extent of gene flow in future environments.

Despite gaps that prevent us from precisely predicting the extent of LD gene flow, the available data on *effective dispersal* and predicted habitat shifts still suggest that these two processes may operate over comparable scales for many tree species. Even within a single reproductive episode, dispersal has the potential to increase genetic variation of local recruits with highly differentiated immigrant genotypes that, depending on the predominant direction of dispersal, could be either pre-adapted to the new local climatic conditions or less adapted than local genotypes. Whether such immigration will enhance or hamper local adaptation upon climate change in subsequent generations will depend on multiple factors, as discussed in the next two sections. In what follows, it should be kept in mind that interspecific variation in the rate and magnitude of LD gene flow, and in other key determinants of population spread rate as time to first reproduction, may substantially affect the variation in the response of forest trees to climate change (Nathan *et al.* 2011; Table 1). In particular, if climate change and other global change processes such as habitat fragmentation disrupted dispersal mutualisms, LD gene flow might be compromised in tree species that are both pollinated and dispersed by animals.

## What are the possible effects of gene flow on adaptation?

We can conceptualise the potential effects of gene flow on adaptation through a simple model of species range evolution, where selection varies both in space and time (Pease *et al.* 1989; Polechova *et al.* 2009; Box 2). Local climate can be thought of as imposing specific selection pressures on a complex set of phenotypic traits (e.g. phenology, frost hardiness, growth and seed size), and defining different optimal trait values through various trade-offs, depending on specific combinations of climatic conditions encountered within the range. As an illustration, in Sitka Spruce, trees originating from higher latitude with lower annual mean temperature cease growing earlier in the season than trees from lower latitude, when grown in common garden (Mimura & Aitken 2007a), suggesting different optimal *bud set* (Glossary) dates along temperature gradients within the range. The simple conceptual model in Box 2 connects adaptation to demography by assuming that an individual’s contribution to population growth declines as it departs from the locally optimal phenotype. This model suggests that gene flow has antagonistic effects on adaptation by modifying the various sources of *genetic load* (Glossary) depressing population mean fitness, and thus population growth (see Bridle *et al.* 2009 for a review). We here review these effects by considering their alternative evolutionary consequences.

**Box 2 A simple conceptual framework illustrating the antagonistic effects of gene flow**

Two closely related theoretical models (Pease *et al.* 1989; Polechova *et al.* 2009) have explored the question of evolution in environments changing both in space and time, mimicking the effects of climate change in species with wide distributions. These models envision a species distributed along some linear environmental gradient, such as the Sitka spruce distributed along a large latitudinal gradient of temperatures (Mimura & Aitken 2007a). We use this empirical example to illustrate potentially realistic values of parameters for the model. We assume fitness depends quadratically on how well an individual tree is adapted to its local ecological conditions, that is, how its phenotype matches the local optimum. By averaging over phenotypes in the local population, one may then write the mean fitness (here the Malthusian population exponential growth rate) in a given location as:

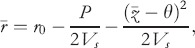

where *r*_0_ is the contribution to population growth of an individual with the optimal phenotype, *V*_*s*_ describes how well individuals that deviate from this optimal phenotype perform (and is thus inversely related to the strength of *stabilising selection*), *θ* is the optimal phenotype in that location, 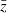 is the local mean phenotype in the population and *P* is the local phenotypic variance around this mean. This expression shows that the mean fitness in a variable population subject to *stabilising selection* is reduced in two ways: (1) *Standing load* (*P*/2*V*_*s*_): caused by phenotypic variation and present even when the mean phenotype matches the optimum; (2) *Evolutionary load* [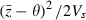]: caused by departure of the mean phenotype from the local optimum. *Evolutionary loads* can be generated by selection that varies over space or time (see Bridle *et al.* 2009 for a review).

Pease *et al.* (1989) and Polechova *et al.* (2009) make specific predictions about how migration might affect the *evolutionary load* in a changing climate. Their models assume the optimum phenotype changes linearly through space, with slope *b*. This is similar to Sitka spruce, where *bud set* date increases linearly with the local mean annual temperature, which itself varies linearly with the distance to the Southern margin of the species range: assuming current *bud set* date corresponds to the optimum, this gives an estimate of *b* such that optimal *bud set* date increases by 13 days every °C, or by 3.24 days per 100 km (Mimura & Aitken 2007a; Aitken *et al.* 2008). Climate change can be approximated by this gradient of optimal phenotypes being constantly shifted through space at rate *v*. According to different climate models, mean annual temperature may increase by 3–5 °C in the generation time of Sitka Spruce (Aitken *et al.* 2008), which gives an estimate of *v* as a shift of *c.* 1000–2000 km per generation. Migration is modelled as a diffusion process, with average distance between parent and offspring *σ*. There are feedbacks between the evolution of the mean phenotype through time and space and that of the population density, mediated through gene flow and the local growth rates (Pease *et al.* 1989; Polechova *et al.* 2009).

Further assuming that genetic variation for the trait under selection is relatively weak and does not vary through space, Pease *et al.* (1989) predict that the loss of fitness at the scale of the range due to *evolutionary load* is:

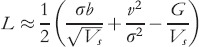

where *G* is the *genetic variance* for the trait. Though this prediction might be crude in the case of forest trees with large within population *genetic variance*, it has heuristic value. Indeed, the first term in parentheses can be interpreted as the component of phenotypic mismatch due to spatial variability in the optimal phenotype and gene flow (*migration load*); this part of the load increases with dispersal distance (*σ*). The second term describes phenotypic mismatch due to the lagging response of the mean phenotype to temporal change in the local optimum (*lag load*). This part of the load decreases with dispersal distance because migration helps the species track its shifting optimum through space. The third term in eqn 2.2 shows that the *evolutionary load* declines with *genetic variance G* because *response to selection* increases. Dispersal distance also affects the evolution of *genetic variance G* (Barton 2001; Polechova *et al.* 2009; Bridle *et al.* 2010), with positive effects on the *evolutionary load* (eqn 2.2), but negative effects on the *standing load P*/2*V*_*s*_ (eqn 2.1).

### Gene flow constrains local adaptation

Because gene flow homogenises allele frequencies across space, high gene flow could constrain *adaptive divergence* (Glossary) along environmental gradients (Garcia-Ramos & Kirkpatrick 1997; but see Barton 2001; Yeaman & Guillaume 2009; Bridle *et al.* 2010 for a revised consideration of the strength of such constraints). Some theoretical models predict in particular that gene flow from large central populations into small peripheral ones may swamp local adaptation in marginal areas, preventing range spread beyond some critical environmental limit (Kirkpatrick & Barton 1997; review in Bridle & Vines 2007). Gene flow then causes *phenotypic clines* (Glossary) for adaptive traits to deviate from their optima (Box 2). Interestingly, the interaction of strong gene flow with selection on multiple traits could result in some *phenotypic clines* being flatter, and some steeper, than optimal because of genetic and selective interactions among traits (Guillaume 2011; Duputié*et al.* 2012). Furthermore, the constraining effects of migration on divergence are predicted to be more severe when divergence involves many loci of small effects rather than few major genes with large effects on the phenotype (Yeaman & Guillaume 2009). In forest trees, the former situation seems to be the most common (Neale & Kremer 2011).

Common garden experiments in forest trees (Box 1) suggest that genotypes can perform poorly when transferred to climates far from their location of origin. Maladaptation of LD migrants could thus reduce the mean fitness in forest tree populations, generating a *migration load* (Glossary). Such *migration load* would be of concern if gene flow is extensive over LDs (see section ‘How far do seeds and pollen disperse in trees?’) and if phenotypic mismatch of immigrants is mostly due to long-lasting genetic effects (Aitken *et al.* 2008). In a Swedish population of *Pinus sylvestris*, Nilsson (1995) indeed found that offspring sired by naturally dispersing pollen had significantly slower growth and higher freezing resistance than expected if offspring were sired only by pollen produced locally. Natural pollination thus resulted in a phenotypic shift corresponding to that expected if most pollen originated from higher latitudes by 1 to 2 degrees (Nilsson 1995).

However, there is little evidence that gene flow has strongly limited adaptation in forest trees in the past. Comparison of genetic differentiation at neutral molecular markers vs. *adaptive traits* (Glossary) repeatedly suggests that extensive gene flow (presumably mostly through pollen dispersal) has not prevented rapid adaptive divergence of extant populations (Savolainen *et al.* 2007; Kremer *et al.* 2010; for theoretical predictions see Kremer & Le Corre 2011). Populations under different climates may however have diverged while still being far from the locally optimum phenotype. Determining how much observed *phenotypic clines* deviate from what would be optimal under local conditions is however difficult to assess, and remains an open issue in evolutionary biology (Barton 2001; Butlin *et al.* 2003). When *provenance tests* (Glossary) have been replicated over a broad range of climatic conditions, *provenance* (Glossary) *reaction norms* (Glossary) allow comparison of optimal and original climate for each population: mismatches are not uncommon and often consistent with a maladaptive effect of gene flow at range margins (Box 1).

### Gene flow enhances the *response to selection*

Natural selection operates by sieving from genetic variation found within populations. Local genetic diversity is therefore the fuel of evolutionary change. Forest tree populations harbour high diversity both for molecular markers and quantitative traits (Hamrick *et al.* 1992), with *heritabilities* (Glossary) typically above 0.4 for wood characteristics or phenological traits such as *bud set* date (Cornelius 1994). The maintenance of such high levels of quantitative variation for traits closely linked to fitness remains a paradox where strong *stabilising selection* (Glossary) is acting to reduce variation within populations (see Johnson & Barton 2005 for a review). Theoretical models predict that increases in *genetic variance* (Glossary) due to gene flow could be substantial (Barton 2001). Along climatic gradients, the increase in *genetic variance* within localities due to gene flow is predicted to be proportional to the change in mean *breeding value* (Glossary) along the typical dispersal distance (Barton 2001). For example, in Sitka Spruce, the *breeding value* for *bud set* date varies by 3.24 days every 100 km while the within-population phenotypic standard deviation of *bud set* date is typically *c.* 10–25 days (Mimura & Aitken 2007a; Aitken *et al.* 2008); LD gene flow over distances of *c.* 100 km would then lead to *heritability* for *bud set* date greater than 0.4 even for relatively strong *stabilising selection*. Interestingly, as genetic variance increases, local populations are better able to respond to local selection, which sharpens genetic clines and thus reinforces the effect of gene flow on genetic variance (Barton 2001; Bridle *et al.* 2010). In addition to the mean dispersal distance, the whole shape of the *dispersal kernel* (Glossary) is predicted to affect the spatial distribution of genetic variation and diversity of migrants (Travis *et al.* 2010), especially in the context of range expansion.

Extensive gene flow in trees is generally thought of as a major explanation for their high within-population diversity (Hamrick *et al.* 1992). Together with the strong selection acting at the juvenile stage experienced by trees, with low survivorship in large, diverse, cohorts of saplings, this may allow rapid adaptation to changing climates. Kelly *et al.* (2003) and Jump *et al.* (2006) report empirical examples of rapid genetic changes in forest trees, however, there is little direct evidence that these rapid responses trace to genetic variation contributed by gene flow. Using a mechanistic model of beech stand dynamics, Kramer *et al.* (2008) predicted little effect of pollen dispersal distance on the evolution of within-stand genetic diversity, but their model ignored the potentially large phenotypic divergence of immigrants (e.g. Nilsson 1995). If gene flow among differentiated populations is a persistent source of genetic variation, there should be strong correlations between *genetic variance* within populations and the amount of heterogeneity in the environment at the regional scale. In a study of 142 populations of lodgepole pine, Yeaman & Jarvis (2006) indeed found that the variance for growth among individuals from the same locality measured in *provenance tests* (Glossary, Box 1) correlated with regional climatic heterogeneity.

### Gene flow facilitates the tracking of environmental change

Assuming an initially locally adapted population, the new selection pressures induced by climate change will cause the optimal phenotype to deviate from the extant mean phenotype, thus creating a *lag load* (Glossary, Box 2). Gene flow will reduce the *lag load* in two different ways: (1) by helping track the shift of the optimum phenotype through dispersal of pre-adapted genotypes found somewhere else in the range (see eqn 2.2 in Box 2) and (2) by augmenting the *response to selection* (Glossary) through a general increase in genetic variation (see previous paragraph). Quite generally, dispersal should help adaptation in environments that are changing both in space and time (Blanquart & Gandon 2011). Beyond theoretical predictions, there is little experimental evidence on the impact of gene flow on the *lag load* of tree populations engaged in an evolutionary race with a changing environment. Simulating the evolution of growth cessation date in pine and birch, Kuparinen *et al.* (2010) found that the large lag that developed after one century of climate warming (*c.* 40 days behind the optimal date) was only moderately reduced (by 2 days) by the higher pollen and seed dispersal distances of birch along the climatic gradient.

### Gene flow affects inbreeding levels

Gene flow may also affect genetic variation for fitness independently from the issue of adaptation to climate, by affecting genetic resemblance between mates. Early *inbreeding depression* (Glossary) is widespread in largely outcrossing species such as trees and inbreeding was found more frequently in isolated marginal populations (Mimura & Aitken 2007b), which may depress their mean fitness and their ability to persist in a changing environment. In many plant species with small isolated populations, progeny formed by crosses between populations show higher fitness than that obtained by within population crosses (e.g. Willi & Fischer 2005). Such a pattern of *heterosis* (Glossary) is however not expected generally very strong in trees, due to the combination of large population size and extensive gene flow (for theoretical predictions, see Glémin *et al.* 2003; Lopez *et al.* 2009). Conversely, small amounts of gene flow between formerly isolated populations can also in theory seriously disrupt fitness due to negative interactions between genes having evolved separately (Edmands & Timmerman 2003). This is aggravated by the fact that isolated populations have evolved in different environments (Frankham *et al.* 2011). Contrasting results from artificial crosses between distant populations, however, fail to provide solid evidence for this type of *outbreeding depression* (Glossary) in trees (Harfouche *et al.* 2000; Goto *et al.* 2011). Because tree populations are seldom isolated from each other, *outbreeding depression* due to negative gene interactions is expected to be rare (Frankham *et al.* 2011).

## How will the different effects of gene flow balance each other in the context of climate change?

We here suggest that the positive effects of gene flow may often dominate negative effects for forest trees confronted to climate change. We conclude this because of the specific life history of forest trees, with high fecundity and intense competition at the juvenile stage. However, this balance of effects is likely to be modulated by (1) the regional context (e.g. expanding edge or retracting part of the range) and (2) the dispersal syndrome (relative strength of pollen vs. seed dispersal).

### Balance between antagonistic effects

**Figure 3:**
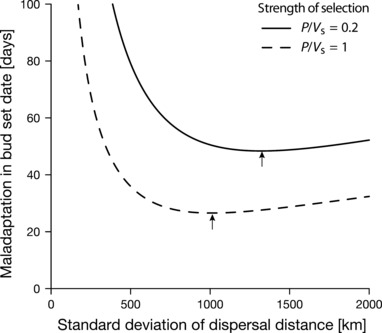
Effect of dispersal distance on the evolutionary load. Maladaptation in bud-set date in Sitka Spruce as a function of dispersal distance in a shifting climate. Maladaptation is predicted as 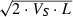 (eqn 2.2, Box 2). Optimal bud-set date varies along a latitudinal gradient of mean annual temperature and here shifts at a constant rate in time. Arrows indicate the optimal dispersal distances minimising maladaptation at the scale of the entire range. Parameter values are given in Box 2. The phenotypic standard deviation of bud-set date is 17 days (Aitken *et al.* 2008). *Heritability* is 0.5, consistently with estimates in other species (Savolainen *et al.* 2004). The strength of *stabilising selection P/V*_*s*_ is 0.2 (continuous line – median value taken from Johnson & Barton 2005) or 1 (dashed line – stronger selection).

Maladaptation in a changing climate is caused by mismatches between optimal and realised mean phenotypes, due to environments that are changing in time and space too fast for the population to adjust to these changes. Such mismatch depresses the mean fitness of populations, generating an *evolutionary load* (Glossary, Box 2). Setting aside the effects of gene flow on *genetic variance*, the simple model in Box 2 predicts that there is an optimal level of migration that minimises such *evolutionary load* under a shifting climate: when dispersal distance is short, the *lag load* decreases fast with increasing migration, which helps the population track the optimal climate (Fig. 3). If gene flow is too high, however, local adaptation is prevented (*migration load*) and maladaptation increases (albeit slowly) with increasing migration (Fig. 3). The optimal dispersal distance is higher if the environment changes more quickly in time in a given location, and if selection varies less sharply in space (Box 2). As an illustration, in the evolution of *bud set* date in Sitka Spruce (Mimura & Aitken 2007a; Aitken *et al.* 2008; see Box 2), the optimal migration distance is relatively large (immigrants should on average originate from locations with mean temperature differing by more than 3 °C to the local site, i.e. more than 1000 km). This suggests that, for a range of realistic dispersal distances, the positive tracking effect of dispersal should dominate its negative effects on local adaptation.

Once the effects of gene flow on the evolution of *genetic variance* are taken into account, the constraining effects of migration on adaptation in marginal populations is much weakened. Very high gene flow seems instead to facilitate adaptation across a wide array of environmental conditions, but at the cost of a reduced fitness everywhere in the range. This cost could ultimately compromise species persistence (for theoretical predictions see Barton 2001; Polechova *et al.* 2009), especially when the stochastic effects of finite populations are included (Bridle *et al.* 2010). The very high fecundity, long life span and strong competition at the juvenile stage, which are characteristic of forest trees, could in principle permit very high *genetic load* causing massive mortality at the juvenile stage, without having much impact on adult density. Further exploration of connections between forest trees population dynamics and genetic diversity are needed to conclude when the demographic cost of adaptation compromises persistence.

Overall, models that integrate different antagonistic effects predict that intermediate levels of gene flow (e.g. between one and 10 migrants per generation) suffice to replenish *genetic variance* eroded by drift and selection, and alleviate *inbreeding depression* without causing large *migration load*, thus maximise mean fitness in heterogeneous environments (Lopez *et al.* 2009; see also Blanquart & Gandon 2011 for the case of spatio-temporal variation). Empirical evidence that migration enhances fitness in marginal habitats of several plant species supports such predictions (Kawecki 2008). We lack similar direct evidence in forest trees. The admixture of genotypes of diverse geographical origin is increasingly thought of as key to successful establishment of introduced populations, because of it increases the *genetic variance* necessary for adaptive responses (see Zheng & Ennos 1999 for an example in introduced Pine populations). Experimental manipulation of gene flow in forest trees would provide valuable data to better understand its constraining or boosting effects on adaptation to local climate.

### Regional variation

Predicted shifts in *bioclimatic envelopes* imply that current populations at the trailing and leading edges of the range will face different adaptive challenges. Southern margin populations will face climatic conditions not encountered anywhere in the current species range. Will these challenged populations have the evolutionary potential (Gomulkiewicz & Houle 2009) to adapt before going extinct? Their persistence will depend on whether the evolutionary or demographic constraints preventing current establishment in warmer or drier climates will be relaxed enough to enable enlargement of species’ realised niches. Conversely, at the northern margins, new areas will become suitable for growth, but the success of colonisation may depend on the genetic make-up of new population founders.

Gene flow can have contrasting consequences for populations at trailing and leading edges of a shifting range (Hampe & Petit 2005): populations at the leading edge or in the central part of the distribution are likely to receive ‘pre-adapted’ genes from more southern populations, and gene flow may facilitate their adaptation (Hu & He 2006). The opposite may be true for populations at the rear end that encounter an entirely novel environment. The flow of pre-adapted genes from central populations is then not possible, which may increase maladaptation and extinction probabilities in populations at the southern margins. However, both demographic and genetic rescue effects of dispersal from larger populations within the species’ range may help those marginal populations to persist. The precise balance of the negative and positive effects of gene flow remains to be explored in this context.

### Balance between the effects of seed and pollen flow

Balance between the negative and positive effects of gene flow may also vary with the relative contribution of seed and pollen dispersal.

Both pollen flow and seed flow contribute substantially to genetic diversity On the one hand, pollen often disperses farther than seeds (see section ‘How far do seeds and pollen disperse in trees?’ and Table 1) and in greater quantities. On the other hand, a single pollen grain carries half the number of alleles compared with a single seed, and only seeds can establish a new population in a remote habitat. Due to long-generation times in trees, migrant seeds accumulate in a new population over years before the new generation reproduces, promoting high levels of diversity in recently founded populations (Austerlitz *et al.* 2000). Long-distance gene flow mediated by pollen in marginal habitats is therefore conditional on the successful establishment of shorter distance migrating seeds.

Selection can be less efficient at removing badly adapted immigrant alleles when their deleterious effects are partly masked in hybrids (Lopez *et al.* 2008). When selection varies sharply in space, pollen dispersal was therefore predicted to generate higher *migration loads* than equivalent seed dispersal (Lopez *et al.* 2008). Selection at the gametophytic stage may further affect these differences (Hu & He 2006). Because, the movement of alleles by pollen necessarily involves combining with existing genetic variation, seed and pollen dispersal could thus have different consequences for population divergence, maintenance of within-population diversity and mean fitness (Lopez *et al.* 2008).

Most models of adaptation and migration in a heterogeneous environment (e.g. Pease *et al.* 1989; Kirkpatrick & Barton 1997; Polechova *et al.* 2009; see Box 2; but see Butlin *et al.* 2003) consider a single dispersal parameter. With pollen and seed dispersal, demographic migration is partially uncoupled from gene flow. Hu & He (2006) predicted that pollen dispersal could slow down or accelerate range expansion in some homogeneous environments by interfering with the spread of beneficial or deleterious mutations. At retracting range margins, seed and pollen dispersal may play very different roles on adaptation: seed dispersal enhances the probability of adaptation in a sink habitat, while pollen dispersal generally compromises it (Aguilée, R., Shaw, F. H., Rousset, F., Shaw, R.G., Ronce, O. unpublished data). Conversely, both pollen flow and seed flow could have positive effects at expanding range margins. In the presence of pollen limitation, LD pollen flow could moreover prevent extinction in marginal populations (Butlin *et al.* 2003).

## Future research directions

### Develop new methods to trace pollen and seeds

Experimental dispersal studies monitoring LD pollen and seed dispersal have often been limited in spatial scale due to (1) overlapping of the *pollen/seed shadows* (Glossary) masking LDD, (2) dilution effect (LDD is rare and requires high power to observe, let alone measure) and (3) large numbers of putative sources (characterising their positions and genotypes is time- and cost-intensive). Using highly polymorphic genetic markers like microsatellites greatly overcomes the first point, and the advent of next-generation sequencing will improve power and resolution; however, it is still necessary to conceive new experimental designs dealing with points (2) and (3). We propose potential strategies here, mostly relying on a stronger interaction with mechanistic approaches.

#### Making use of meteorological data

For wind-mediated gene flow, available weather data could help determine the potential range of pollen and seed dispersal within particular landscapes, regions, or continents. Such an analysis requires regional meteorological datasets, phenological observations over a wide region and sufficient understanding of the meteorological factors driving pollen and seed emission and spread. Products of regional and global weather reanalysis, such as the North American Regional Reanalysis (NARR) dataset and the European Centre for Medium Range Weather Forecast (ECMWF) data, offer useful observational and model-based information on wind, temperature, humidity, radiation and other meteorological data (Schueler *et al.* 2005). On-line interfaces for weather and radiation simulation tools can also be used to evaluate conditions across large dispersal ranges (Bohrerova *et al.* 2009). For pollen, phenological data are available from pollen monitoring networks (e.g. the European Aerobiology Network, EAN) or from phenological observations (e.g. the European Phenological Network). Model-driven weather reconstructions (Solomon *et al.* 2007) can provide estimates of dispersal potentials in past and future climates (Kuparinen *et al.* 2009; Nathan *et al.* 2011). Improved characterisations of wind dispersal mechanisms accounting for interactions between pollen/seeds and turbulent winds in relation to weather conditions can be combined to determine annual and multiannual wind-driven pollen and seed dispersal patterns throughout large geographic regions (Muñoz *et al.* 2004; Thompson & Katul 2008; Nathan *et al.* 2011).

To experimentally trace pollen or seed movement at the continental scale, a joint use of weather data, weather-forecasting models and field observation of pollen/seed pools seems most promising. Large-scale spatial characterisation of presence/absence of a species, phenology, and airflows were already used to identify temporal windows ideal for LDD and relate them to the actual presence of pollen grains in physical captors (Siljamo *et al.* 2008). A next step would be to measure the diversity of origins in the effective pollen pools through the genetic and/or phenotypic diversity of the seed produced (Nilsson 1995). For species pollinated and/or dispersed by animals, wind circulation patterns may also help in predicting long-range movement of their vectors, especially in combination with recent developments in animal-tracking with microtransmitters and radar (Felicísimo *et al.* 2008; Bridge *et al.*2011). Ultimately, these approaches should provide information not only about the expected rates of LD propagule transport, but also on the most likely original habitats of immigrants, and thus on their expected effect on local adaptation (see section ‘How will the different effects of gene flow balance each other in the context of climate change?’).

#### Taking advantage of adequate landscape configurations

**Figure 4:**
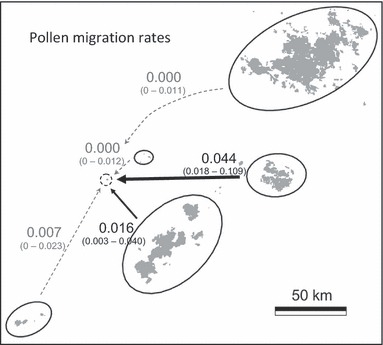
Long-distance effective pollen dispersal of *Pinus sylvestris* L. Estimated effective pollen immigration rates into a *Pinus sylvestris* remnant (encircled with dashed line) from five long-distant populations (encircled with continuous lines) in Central Spain, obtained using maximum-likelihood genetic mixture analysis combined with Monte Carlo assessment of small parameter uncertainty. Continuous (resp. dashed) arrows indicate pollen immigration rates significantly (resp. non-significantly) different from zero. 95% confidence intervals between brackets (modified from Robledo-Arnuncio 2011 with permission from the Publisher).

*Genetic assignment* (Glossary) methods linking pollen, seeds or seedlings to candidate parental populations could be used to evaluate the effective rate and range of contemporary gene flow among discrete populations (Manel *et al.* 2005). Focus could be placed initially on isolated populations or trees, particularly informative about LDD because they are less subject to dilution effects. A recent study using *genetic assignment* in such demographic setting has revealed effective pollen gene flow over 100-km distances in a wind-pollinated species (Fig. 4). For species with extremely low densities, even *parentage analysis* (Glossary) may prove efficient in detecting LD gene flow (Ahmed *et al.* 2009). Female plants, male-sterile or self-incompatible isolated individuals might prove useful traps for investigating the composition of LD effective pollen clouds, and could be distributed at specific positions during the pollination period, for example, using potted plants, flowering branches kept alive or flowering grafts. High-precision aerial photographs and satellite images could be used to retrieve all potential sources at the regional scale and avoid biases due to ghost populations.

An alternative solution, not requiring trap plants but not assessing *effective dispersal* directly, is to characterise the genetic content of the pollen pool by genotyping single pollen grains (Matsuki *et al.* 2007), sampled in volumetric traps from existing *aerobiology* (Glossary) networks or placed at specific sites in a landscape. Ultimately, dispersal inferences from adequate landscape configurations could enlarge the documented range of dispersal especially for abundant species, in which dilution effects complicate the detection of LDD events in general. Note, however, that the atypical demographic conditions of isolated trees that facilitate LDD assessment may result in observed LDD patterns difficult to generalise, for which modelling approaches may be necessary.

#### Combining mechanistic and genetic models

The ability of mechanistic approaches to assess dispersal across multiple scales (see Nathan *et al.* 2008) complements the problematic extrapolation of genetic methods beyond the small scale in which individuals were sampled (e.g. Niggemann *et al.* 2011). Genetic methods, in turn, can provide data to validate mechanistically derived kernels, and to add the required (and often hard to measure) component of post-dispersal establishment effects needed to assess *effective dispersal*. Steinitz *et al.* (2011) have recently combined a mechanistic wind dispersal model to assess seed dispersal, with parentage analysis based on polymorphic molecular markers to assess the ultimate effective dispersal: adults of next generation. Notwithstanding the importance of this work, a formal inferential framework integrating the complementary features of mechanistic and genetic approaches for any dispersal vector is yet to be developed. Future developments in this front should attempt to incorporate mechanistically derived propagule transport probability functions (e.g. as in Kuparinen *et al.* 2009; Nathan *et al.* 2011) into the usual probabilistic contemporary migration rate estimation procedures based solely on genetic likelihoods (Manel *et al.* 2005), allowing an informative joint estimation of LD migration rates and mechanistic parameters affecting them.

#### Developing the connectivity network

Current research on spatial patterns of dispersal and gene flow is dominated by the *dispersal kernel* concept, which bears significant disadvantages when applied to broad scales. Studies using *dispersal kernels* generally require sampling intensities that become unfeasible over LDs. Moreover, they often assume isotropy (i.e. the same *dispersal kernel* for all directions); although this assumption is unrealistic for many systems in which the dispersal vector moves in a directional manner, such as many seasonal winds, downward flow of rivers, and oriented movement of animals. Similarly, *genetic assignment* methods for migration rate estimation typically incorporate neither directional nor other kinds of spatial information. *Lagrangian dispersal* (Glossary) simulations can account for dispersal anisotropy by incorporating turbulence patterns (Bohrer *et al.* 2008), and hourly, daily or seasonal variation in wind direction (Wright *et al.* 2008); this computationally intensive approach, however, is practically limited to relatively short-term small-scale applications. An alternative approach, *connectivity maps* (Glossary), depicts dispersal probabilities between sites based on large-scale datasets and/or models available for the primary dispersal vector, for example, to assess wind connectivity of plants among islands in the southern oceans (Muñoz *et al.* 2004). In principle, this method could be adjusted to many plant species in a variety of spatial scales, if the patterns of movement of the dispersal vector can be estimated. For example, movement rules of animals, and thereby connectivity maps of animal-dispersed plants, can be defined by analyzing paths of GPS-tracked individuals in relation to landscape features measured at one or multiple scales (Cushman 2010). High-resolution wind data collection techniques (Felicísimo *et al.* 2008) and radio and satellite animal tracking (Bridge *et al.* 2011) are also promising for this purpose. Because some key vectors such as wind, inland water systems, ocean currents and migrating birds disperse many plant species, efforts to develop vector *connectivity maps* could advance the study of gene flow via pollen and seeds for a large number of species.

### Implement new experimental approaches to assess evolutionary changes in trees

Despite obvious biological constraints in trees, we recommend setting up experiments that would allow assessing evolutionary changes over successive generations. Existing multisite *provenance tests* constitute in this respect a precious source of data, allowing to assess the adaptive response of different populations across different environmental conditions (Box 1). Although valuable, we suggest implementing additional experiments to overcome the limitations of provenances tests. Indeed, most tests were usually established under ecological conditions that do not encompass predicted climatic changes. They were installed under peculiar experimental settings (plantations and regular spacings) that underestimate the impact of natural selection. Provenances sampled were quite often limited to selected stands for seed production stands and often excluded provenances from marginal sites. Finally, provenance experiments are mostly designed to assess adaptive divergence but less suited to measure evolutionary changes of the same populations along successive generations (i.e. allochronic changes). We therefore suggest initiating new experiments avoiding these shortcomings. For example, allochronic monitoring of evolutionary changes can be assessed in the case of forest stands originating from transferred populations. Artificial transfers have been done in the past in forest trees and some of them are historically well documented (Fallour-Rubio *et al.* 2009). In some cases, these transfers actually mimicked climate changes, as populations were moved from cooler to milder climates (Daubree & Kremer 1993). They would allow to measure evolutionary changes at contemporary times scales, as only a few generations have elapsed since their installation. Additional experiments aiming at monitoring phenotypic changes *in natura* consist in assessing variation of adaptive relevant traits at various filtering stages and in particular from seed to the seedling stages when natural selection is strongest under natural regeneration in forest stands.

Finally, in addition to experiments conducted in the field (in provenance tests or *in natura*), we suggest initiating experiments under controlled conditions mimicking climate change using open top chambers, where the level of CO_2_, temperature and rainfall can be manipulated. Ideally, such experiments should be conducted for short-generation species (willow, birch or poplar) allowing to manipulate as well gene flow among different populations. Selection experiments on short rotation species would not only allow one to monitor genetic changes for key adaptive traits (bud burst and bud set, resistance to drought) that are undergoing strong selection under climate change. They would also give access to the rate and direction of change as a function of standing variation and variation due to gene flow, under a wide range of controlled selection regimes.

### Develop integrative theoretical approaches

#### Extend evolutionary models of adaptation to climate change to the case of trees

Analytical models (e.g. Box 2) provide conceptual insights into how gene flow, adaptation and biotic interactions shape species ranges in stable or changing environments (Pease *et al.* 1989; Kirkpatrick & Barton 1997; Barton 2001; Polechova *et al.* 2009). Available models, however, rarely incorporate salient features of tree life cycles, such as distinct dispersal modes, overlapping generations, or *fat-tailed dispersal kernels*, which may profoundly affect their evolutionary responses to climate change. How the pace of adaptation in a changing environment depends on variation in fitness expressed before vs. after sexual maturity and on the correlation between juvenile vs. adult traits is for instance an important area for future research.

Exploring the effects of LD gene flow on adaptation also requires modelling dispersal as a more complex process than the simple homogeneous diffusion considered in the models summarised in Box 2. What is the evolutionary impact of rare LD dispersal events well beyond the average dispersal distance? Although the effect of *fat-tailed dispersal kernels* on rates of expansion (Clark *et al.* 2003; Thompson & Katul 2008) and neutral diversity (Travis *et al.* 2010) have been explored, we lack similar theoretical investigation of their effects on adaptive diversity in the context of climate change. Answering this question would also help identify critical features of pollen and seed dispersal distributions on which empirical estimates should focus.

The idea that climate change is equivalent to simple spatial shift of local climatic conditions is also a gross simplification. Rather, climatic change may result in new combinations of precipitation patterns, temperature, photoperiod and biotic conditions that occur nowhere within the current range, imposing entirely new selection pressures and favouring the assembly of novel genotypes (Williams & Jackson 2007). Adaptation to climate change may thus require the production of new phenotypic combinations. Reaching such combinations means that natural selection is acting on multiple traits simultaneously; and unfortunately, evolutionary models have often been limited to single traits. Multivariate adaptive responses depend on the amount of *genetic correlation* (Glossary) among traits, which may limit or accelerate adaptation to climate change (e.g. Etterson & Shaw 2001). Modelling those responses as a univariate rather than multivariate process, as done so far, might thus fail to provide an accurate picture of species’ adaptive capacities. Although we have begun to incorporate multivariate evolution into models of migration–selection balance (Guillaume 2011; Duputié*et al.* in review), empirical data are direly missing on patterns of *genetic correlations* among key ecological traits in trees and on the spatial and temporal variation of their joint selection pressures.

Efforts should be made to fill these gaps and help calibrate models with real data, to ultimately be able to merge evolutionary approaches with niche- and process-based ecological forecasting of climate-induced range shifts.

#### Use integrative simulation platforms

Trait-based, mechanistic models have recently been developed enabling predictions of species ranges under current and future non-analogous climates (e.g. Morin *et al.* 2008). For instance, the Phenofit model (Chuine & Beaubien 2001) predicts tree distributions based on existing phenological responses to local climate, drought and frost tolerance. Microevolutionary phenomena described above have only started to be incorporated in such ecological forecast models (Kuparinen *et al.* 2010). There is therefore an urgent need to incorporate genetic and ecological concepts into integrated models to accurately predict the impact of environmental changes on species persistence over the next century and at the continental scale. Efforts should be dedicated to foster development of integrated computer simulation platforms with this aim. Individual-based, population and quantitative genetics simulation packages already exist (e.g. *Nemo*, Guillaume & Rougemont 2006; e.g. *Metapop*, Kremer & Le Corre 2011) that could be extended to include the ecological and spatially explicit layers needed.

A key aspect of the modelling approach advocated here is the overlay of predictions from different processes; ecological niche and *bioclimatic envelope* modelling, variation of gene flow over geographical ranges and evolutionary adaptation of local populations. The basal layer, the climatic layer, defines how changes in climatic conditions over the species’ geographical range modify the localisation of suitable habitats (Thuiller 2003). The second layer describes spatial variation of pollen and seed dispersal and should integrate information from the climatic layer to model the changes of seed and pollen movements caused by climate change through modification of the *dispersal kernels* (Kuparinen *et al.* 2009; Nathan *et al.* 2011), pollen viability (Bohrerova *et al.* 2009), or the timing of pollination and female receptiveness. The third layer integrates information from the two previous ones to predict how local populations adapt to their shifting conditions (e.g. Kuparinen *et al.* 2010). Information from the climatic layer will set the strength of selection acting on different *adaptive traits* by indicating how far from its local optimum a population might be. Information on gene flow from the second layer will indicate hybridisation rates and fitness effects, depending on the geographical origin of the migrants (Savolainen *et al.* 2007; Lopez *et al.* 2008; Yeaman & Guillaume 2009). It will also indicate the potential for colonisation of new habitats. Finally, the outcome of local adaptation can be interpreted in terms of growth and persistence of local populations and how this feeds back into predictions of the intensity of gene flow over larger geographical scales.

## Conclusion

Although much emphasis has been placed on the ability of tree populations to migrate fast enough in response to climate change, we have here examined the potential consequences of LD gene flow on their adaptive response to climate change.

Many tree species have evolved dispersal syndromes enabling the effective flow of genetic information across distant populations inhabiting contrasting environments. We have argued how such exchanges, although potentially maladaptive in some evolutionary and demographic scenarios, may in the case of forest trees favour adaptation to changing climatic conditions, compensating for their long-generation time. However, we are still missing adequate tools to track LD pollen and seed dispersal enabling to predict population responses to changing environments. The demographic impact of phenotypic mismatch in forest trees also remains to be quantified. Our understanding of the interaction between gene flow and local adaptation under realistic ecological, demographic and dispersal assumptions is still limited, and we have suggested potential theoretical and experimental avenues of research for the integration of dispersal biology, ecology and evolutionary quantitative genetics in a better predictive inferential framework.

## Glossary

**Adaptive divergence**: the differentiation among the mean phenotypes of populations subject to different selective pressures.

**Adaptive trait**: a phenotypic trait that enhances the fitness of an individual in a particular environment. Examples for trees are the time of flushing and bud set, and physiological traits determining water use efficiency.

**Aerobiology**: the study of airborne organisms and organic particles.

**Bioclimatic envelope**: the predicted potential geographic distribution of a species in a particular climatic scenario.

**Breeding value**: part of an individual’s phenotype that can be transmitted to its offspring through the transmission of genetic material.

**Bud set**: formation of a terminal bud at the end of the vegetative period, the timing of which is heritable and determines cold tolerance in boreal and temperate trees.

**Connectivity map**: a map describing the cost or the probability of dispersal along possible trajectories linking a set of locations.

**Dispersal kernel**: a probability density function of dispersal distances or locations from a source point.

**Effective dispersal**: dispersal leading to successful establishment or reproduction.

**Evolutionary load**: the mean fitness loss in a population produced by the deviation of the mean phenotype from the local optimum due to varying selection in space and time.

**Fat-tailed dispersal kernel**: dispersal kernel with a slow probability decrease at long distances relative to a negative exponential.

**Genetic assignment**: the probabilistic ascertainment of the original population of an individual genotype.

**Genetic correlation:** non-independent genetic variation for two phenotypic traits, which can be due in particular to the fact that the same genes affect variation of several traits, or that genes affecting different traits do not segregate independently in gametes.

**Genetic load**: the loss of mean fitness in a population due to the departure of individual phenotypes from the optimum in a given environment.

**Genetic variance**: part of the total phenotypic variance that is due to genetic differences between individuals.

**Heritability**: the proportion of phenotypic variation among the individuals of a population in a particular environment that is due to genetic variation.

**Heterosis**: the higher fitness of progeny obtained through crosses between populations rather than within the same population.

**Inbreeding depression**: reduced fitness of inbred individuals.

**Lag load**: the loss of mean fitness in a population due to the lagging response of the phenotypic mean to temporal changes in the optimum.

**Lagrangian dispersal model**: a mathematical description of the trajectories of individual dispersers.

**Migration load**: the contribution of immigrant genes to genetic load (see genetic load).

**Outbreeding depression**: reduced fitness of individuals born to parents from different populations.

**Parentage analysis**: the probabilistic determination of the parents of an individual, frequently using genetic markers.

**Phenotypic cline**: a continuous change of a phenotypic trait along an environmental and/or geographical gradient.

**Pollen/seed shadow:** the density of pollen grains/seeds dispersed at different distances from an individual. It equals the product of the dispersal kernel by the individual’s fecundity.

**Provenance**: the original geographic source of a population or group of individuals (used also to refer to such a population or group).

**Provenance test**: a common garden experiment, in one or more locations, where the genetic variation of different provenances is evaluated (see Provenance).

**Reaction norm**: the set of phenotypes expressed by a particular genotype under a range of environments.

**Response to selection**: the difference between the mean phenotype of the offspring of a group of selected parents and the mean phenotype of the population before selection.

**Stabilising selection**: selection that favours intermediate over extreme phenotypes.

**Standing load**: the loss of mean fitness in a population due to the phenotypic variance around the mean phenotype. Present even when the mean phenotype matches the optimum.
